# Exposure to blue light stimulates the proangiogenic capability of exosomes derived from human umbilical cord mesenchymal stem cells

**DOI:** 10.1186/s13287-019-1472-x

**Published:** 2019-11-28

**Authors:** Kun Yang, Dong Li, Meitian Wang, Zhiliang Xu, Xiao Chen, Qiao Liu, Wenjie Sun, Jiangxia Li, Yaoqin Gong, Duo Liu, Changshun Shao, Qiji Liu, Xi Li

**Affiliations:** 1 Key Laboratory of Experimental Teratology, Ministry of Education Department of Medical Genetics, School of Basic Medical Sciences, Shandong University, 44 Wen Hua Xi Road, Jinan, Shandong 250012 People’s Republic of China; 2 Cryomedicine Laboratory, Qilu Hospital of Shandong University, Jinan, 250012 Shandong China; 3 Stem Cell and Regenerative Medicine Research Center of Shandong University, Jinan, 250012 Shandong China; 4 State Key Laboratory of Crystal Materials, Shandong University, Jinan, Shandong 250100 People’s Republic of China; 5 The First Affiliated Hospital of Soochow University and State Key Laboratory of Radiation Medicine and Protection, Institutes for Translational Medicine, Soochow University, Suzhou, 215123 Jiangsu China; 6 Advanced Medical Research Institute, Shandong University, Jinan, 250012 Shandong China

**Keywords:** Mesenchymal stem cells, Exosomes, Angiogenesis, Light exposure, microRNAs

## Abstract

**Background:**

The therapeutic potential of mesenchymal stem cells (MSCs) may be attributed partly to the secreted paracrine factors, which comprise exosomes. Exosomes are small, saucer-shaped vesicles containing miRNAs, mRNAs, and proteins. Exosomes derived from human umbilical cord mesenchymal stem cells (hUC-MSCs) have been reported to promote angiogenesis. However, the efficacy of exosome-based therapies is still limited both in vitro and in vivo. The present study aimed to develop a new optical manipulation approach to stimulate the proangiogenic potential of exosomes and characterize its mechanism underlying tissue regeneration.

**Methods:**

We used blue (455 nm) and red (638 nm) monochromatic light exposure to investigate the processing of stimuli. Exosomes were prepared by QIAGEN exoEasy Maxi kit and confirmed to be present by transmission electron microscopy and immunoblotting analyses. The proangiogenic activity of blue light-treated human umbilical vein endothelial cells (HUVECs), when co-cultured with hUC-MSCs, was assessed by EdU (5-ethynyl-2′-deoxyuridine) incorporation, wound closure, and endothelial tube formation assays. The in vivo angiogenic activity of blue light-treated MSC-derived exosomes (MSC-Exs) was evaluated using both murine matrigel plug and skin wound models.

**Results:**

We found that 455-nm blue light is effective for promoting proliferation, migration, and tube formation of HUVECs co-cultured with MSCs. Furthermore, MSC-Exs stimulated in vivo angiogenesis and their proangiogenic potential were enhanced significantly upon blue light illumination. Finally, activation of the endothelial cells in response to stimulation by blue light-treated exosomes was demonstrated by upregulation of two miRNAs, miR-135b-5p, and miR-499a-3p.

**Conclusions:**

Blue (455 nm) light illumination improved the therapeutic effects of hUC-MSC exosomes by enhancing their proangiogenic ability in vitro and in vivo with the upregulation of the following two miRNAs: miR-135b-5p and miR-499a-3p.

**Graphical abstract:**

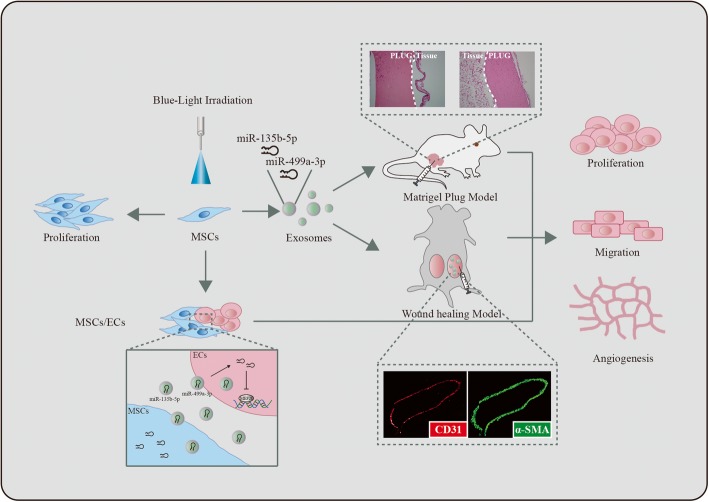

## Highlights

- MSCs constitutionally express opsins for light responsiveness
- Blue light promotes proangiogenic ability of MSC-Exs in vitro and in vivo
- Upregulation of miR-135b-5p/miR-499a-3p-MEF2C signaling in the light-stimulated angiogenesis

## Background

Mesenchymal stem cells (MSCs) are among the most widely studied multipotent stem cells. MSCs have been suggested as promising candidates for a variety of therapeutic applications in various degenerative and inflammatory disorders [1]. In addition to the capacity to differentiate into various cell lineages, MSCs secrete paracrine factors that have been considered to play a critical role in tissue regeneration [2]. Cell-derived exosomes are emerging as a new mechanism in intercellular communication [3]. Exosomes are extracellular vesicles formed by the fusion of vesicular bodies with the plasma membrane [4]. It has been reported that exosomes derived from MSCs are enriched in mRNAs or microRNAs (miRNAs) and their therapeutic effects in myocardial ischemia, acute kidney injury, and liver fibrosis have been previously investigated [5–8]. Importantly, MSC-derived exosomes (MSC-Exs) have emerged as a highly promising therapeutic tool because of their reduced immunogenicity and increased tissue regeneration ability through the promotion of angiogenesis and induction of cell proliferation [9, 10].

Angiogenesis, the growth of blood vessels from pre-existing blood vessels and subsequent expansion of the blood vessel network is crucial in tissue regeneration [11]. Endothelial cells (ECs) are the primary constituents of new vessels, and many functions of ECs are required for angiogenesis. However, exosome-based therapies to stimulate EC angiogenesis in clinical practice are still impeded by some issues, such as limited efficiency and uncharacterized molecular mechanisms [12, 13]. Light is an invaluable tool for manipulating cell behavior by opsin-triggered phototransduction and/or thermal energy supply in living cells [14]. Development of a non-invasive, light-driving approach to enhance the efficiency of exosome-based therapies will be valuable for tissue regeneration. In contrast to the vast amount of information available related to improving the proangiogenic potential of MSC-Exs by combining MSCs with biomaterials, only limited data are available regarding the response of MSCs to light stimulation and about the functions of exosomes that promote angiogenesis of ECs. Therefore, it is essential to develop new optical manipulation techniques to enhance the range of the proangiogenic capabilities of MSC-Exs.

Furthermore, it has been reported that exosomes enhance angiogenesis by delivering microRNAs, mRNAs, and/or protein molecules [1, 15]. We have reported that miR-135-5p and miR-499a-3p, derived from serum exosomes, jointly promote the proliferation and migration of ECs by regulating a single common target gene, *MEF2C* (myocyte enhancer factor 2C) [16]. However, the mechanism underlying optical stimulation enhancement of exosome angiogenesis efficiency is not well characterized.

In the present study, we first characterized human umbilical cord mesenchymal stem cells (hUC-MSCs) and their constitutional expression of blue- and red-sensitive opsins, which are the photoreceptors present within mammalian retina and skin [17]. Next, we used blue (455 nm) and red (638 nm) monochromatic light exposure to investigate the processing of stimuli that preferentially trigger proliferation and migration of ECs. Our results demonstrated that illumination with 455-nm blue light could stimulate the proangiogenic potential of hUC-MSCs both in vitro and in vivo. Moreover, the elevated levels of miR-135b-5p and miR-499a-3p due to blue light exposure increased proangiogenic capacities in both MSCs and MSC-Exs. Therefore, our optical modulation method is expected to provide a promising platform to trigger angiogenesis both in vitro and in vivo for tissue regeneration.

## Methods

### Cell culture, qRT-PCR, and immunological procedures

The human umbilical cord mesenchymal stem cells (hUC-MSCs) were described previously [18]. The study has been approved by the Ethics Review Committee for Human Studies of the Shandong University Qilu Hospital. The hUC-MSCs were cultured in α-MEM medium supplemented with 10% exosome-free fetal bovine serum (Cellmax, Beijing, China) and four factors: VEGF (2 ng/mL), bFGF (2 ng/mL), EGF (2 ng/mL), and PDGF-BB (2 ng/mL) (Proteintech, Rosemont, IL, USA) in 95% air/5% CO_2_ at 37 °C. Cells of passages 2 to 4 were used. Human umbilical vein endothelial cells (HUVECs) were purchased from ATCC and were described previously [16].

Real-time quantitative RT-PCR (qRT-PCR) analysis was performed using an ABI 7500 System (Applied Biosystems, Foster City, CA). The reverse transcription primers and the primer sets specific for amplification of miR-135b-5p and miR-499a-3p were described previously [16]. Antibodies against the following proteins were purchased: anti-OPN4 (ab19383, polyclonal antibody produced in rabbit, Abcam, Cambridge, UK), anti-OPN1SW (DF10234, polyclonal antibody produced in rabbit, Affinity, Cincinnati, USA), anti-RRH (AF9153, polyclonal antibody produced in rabbit, Affinity, Cincinnati, USA), anti-RHO (DF5046, polyclonal antibody produced in rabbit, Cincinnati, Santa Cruz, USA), anti-MEF2C (SC13266, polyclonal antibody produced in goat, Santa Cruz Biotechnology, Santa Cruz, USA), anti-HSP70 (ab181606, monoclonal antibody produced in rabbit, Abcam, Cambridge, UK), anti-CD9 (ab92726, monoclonal antibody produced in rabbit, Abcam, Cambridge, UK), anti-CD31 (GB11063, polyclonal antibody produced in rabbit, Servicebio, Wuhan, China), and anti-α-SMA (GB13044, monoclonal antibody produced in mouse, Servicebio, Wuhan, China). Immunoblotting, immunofluorescence staining, and immunohistochemistry examination were performed as described previously [16].

### Photostimulation systems

hUC-MSCs (2 × 10^5^ cells/mL) were exposed to a 455-nm blue light-emitting diode (LED) or 638-nm red LED light (Yuanming Lasever, Ningbo, China), at a distance of 12 cm from the LED light source. The irradiation duration was 45, 60, 90, or 120 min daily over three consecutive days at room temperature. The full power density of LED irradiated onto the cells was 300 μW/cm^2^, and the power density could be reduced to 180 or 100 μW/cm^2^. Time-matched control cells were kept in the dark during the same time points.

### EdU incorporation and migration assays

EdU (Cell-Light™ EdU Cell Proliferation Detection Kit, RiboBio, Guangzhou, China) was added at a concentration of 100 μM, and the cells were cultured for an additional 2 h. After removal of the EdU-containing media, the cells were fixed with 4% paraformaldehyde at 25 °C for 30 min, washed with glycine (2 mg/mL) for 5 min in a shaker, treated with 0.2% Triton X-100 for 10 min, and washed twice with PBS. Click reaction buffer (Tris-HCl, pH 8.5, 100 mM; CuSO_4_, 1 mM; Apollo 550 fluorescent azide, 100 μM; ascorbic acid, 100 mM) was then added. After 20 min, the cells were washed three times with 0.5% Triton X-100, stained with 4′,6-diamidino-2-phenylindole (DAPI) for 10 min at room temperature, washed five times with 0.5% Triton X-100, and finally, immersed in 150 μL PBS and examined under a fluorescence microscope. The cell migration ability was tested with in vitro wound closure assays as described previously [16].

### Endothelial tube formation assay

Endothelial tube formation assays were performed according to the manufacturer’s protocol (BD Biosciences, Franklin Lakes, NJ, USA). Matrigel (80–100 μL) was added to a 96-well microtiter plate and allowed to polymerize. HUVECs (2 × 10^4^ cells/mL) were plated on the Matrigel. After incubating for approximately 2–4 h at 37 °C, the cells were observed under a microscope (Olympus BX41) and photographed. Tube length was measured using ImageJ software.

### Exosome purification and characterization

After culturing the hUC-MSCs for 48–72 h in serum-free α-MEM medium with four factors (2 ng/mL/each factor): VEGF, bFGF, EGF, and PDGF-BB, the supernatant was collected. Exosomes were isolated using a exoEasy Maxi kit (QIAGEN GmbH, Hilden, Germany) according to the manufacturer’s protocol. The presence of exosomes was verified using the exosomal markers CD9 and HSP70. Purified exosomes were identified by transmission electron microscopy. A drop of exosomes (20 μL) was transferred to a covered copper mesh and allowed to sit at room temperature for 10 min before excess liquid was removed with a piece of filter paper. The coated copper mesh was transferred to a 3% glutaraldehyde fixative droplet and kept at 25 °C for 5 min before excess liquid was removed with a piece of filter paper. The coated copper mesh was then washed with distilled water ten times. The coated copper mesh was then transferred to a 4% uranyl acetate dye solution droplet and allowed to sit at 25 °C for 10 min before excess liquid was removed with a piece of filter paper. The coated copper mesh was then transferred to 1% methylcellulose droplets and allowed to stand at 25 °C for 5 min before excess liquid was removed with a piece of filter paper. After allowing the coated copper mesh to naturally dry, the sample was analyzed by transmission electron microscopy.

### Animal model fabrication of deep second-degree burn

Animal care and experiments were performed in accordance with the protocols approved by the Animal Care and Use Committee of Shandong University School of Basic Medical Sciences. Experiments were performed on male C57BL/6 mice (6~8 weeks old, from Model Animal Research Center of Shandong University, Jinan, China). Animals were housed for at least 7 days prior to experiments in a ventilated and temperature-controlled room and had access to water ad libitum. Anesthesia was performed by intramuscular injection of 1% pentobarbital sodium (50 mg/kg). The hair on the dorsal skin of the mice was removed by electric clippers. A 1-cm-diameter hollow plastic tube was placed to the back of the mice, and 2 mL boiling water (97~100 °C) was quickly injected with the preheated syringe into the plastic tube and applied to the skin for 25 s. During the process of thermal injury, the temperature of the hot water decreased by less than 2 °C. Two burn zones were created on each half of the dorsal skin. A deep second-degree burn injury resulted from this procedure, and the burn depth was confirmed by pathology. For the control group, the boiling water was replaced with distilled water at room temperature.

### Animal grouping, treatment, and tissue histology

The C57BL/6 mice were randomly divided into three time points (four mice at each time point) to assess post-burn endpoints at 3, 5, and 7 days. The mice in the treatment group were injected with exosomes (100 μg of MSC-Exs with/without monochromatic blue light illumination suspended in 200 μL PBS) subcutaneously at different four sites on each burn zone immediately after the burn. Those in the control group were injected with 200 μL PBS only. The animals were housed individually. At 3, 5, and 7 days after the burn occurred, the mice were sacrificed by isoflurane anesthesia, and the wound area was collected for further analysis.

The excised skin samples were fixed in 10% formalin, dehydrated in graded alcohol, and embedded in paraffin for histological examination. The 4-μm serial cross sections were subjected to hematoxylin and eosin staining and immunofluorescence staining. Hematoxylin and eosin (H&E) staining was performed according to standard procedures. For the immunohistochemistry analyses, CD31 and α-SMA were detected using immunofluorescence staining. As in the procedures described, 4-μm sections were rehydrated and boiled in citrate sodium buffer for 15 min for antigen recovery, and then incubated with 5% donkey serum in PBS at 37 °C for 60 min. The sections were incubated with CD31 (1:200, Servicebio, Wuhan, China) and α-SMA (1:200, Servicebio, Wuhan, China) antibodies overnight at 4 °C. The sections were then incubated with the secondary antibodies Alexa Fluor 488 (1:200, Abcam, Cambridge, UK) and Alexa Fluor 594 (1:200, Proteintech, Rosemont, IL, USA) for 60 min at 37 °C. Counterstain with DAPI and mounted on glass slides. Microscopic observation and photograph were performed using a confocal microscope (PerkinElmer Opera Phenix High Content Screening System).

### Statistical analysis

The data are presented as mean ± standard deviation. All tests were performed as two-sided, and a significance level of *P* < 0.05 was considered to indicate statistical significance (*P < 0.05, **P < 0.01, ***P < 0.001, and ****P < 0.0001). GraphPad Prism 5.0 (GraphPad Software Inc., San Diego, CA, USA) was used for all statistical analyses. All photographic images of western blots, Edu incorporation assays, migration assays, and immunohistochemical staining are representative of at least three independent experiments.

## Results

### Characterization of photoreceptors for light irradiation of MSCs

**Fig. 1:**
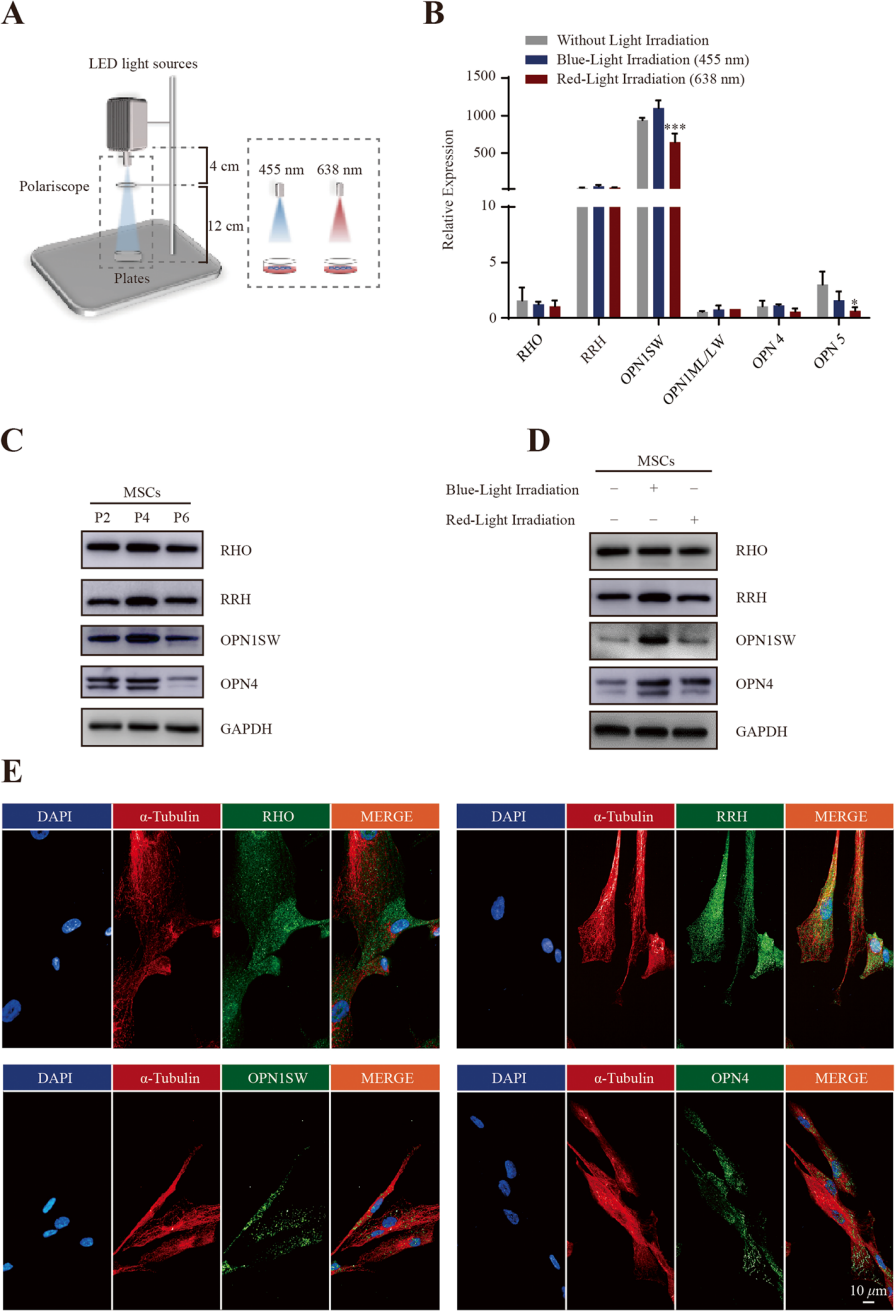
Characterization of MSC photoreceptors for blue/red light irradiation. **a** Schema of the LED photosystem. **b** Real-time PCR screening of the basal and stimulated expression of opsins as indicated (*OPN4* constitutional expression level was selected as the control group, fold-change = 1). **c** Proteins were extracted from P2, P4, and P6 passages of MSCs and subjected to immunoblotting. **d** Proteins were extracted from P4 passage of MSCs with blue/red light illumination and subjected to immunoblotting. **e** Immunofluorescence staining of the indicated photoreceptors in MSCs

To explore whether light exposure affects the phenotype and function of MSCs, we used a monochromatic photosystem illustrated in Fig. 1a to evaluate the response of MSCs to the blue (455 nm)/red (638 nm) LED light. The blue light has no overlap of spectrum wavelength with the red light.

To test whether MSCs express photoreceptors that could respond to light stimulation, real-time PCR was first used to explore the basal and irradiated level of the photopigment molecules in MSCs [19]. As shown in Additional file 1: Table S1, which lists the identified human opsins, gene expression of *RHO*, *RRH*, *OPN1SW*, *OPN4*, and *OPN5* was determined (Fig. 1b). These opsins serve as a photoreceptor response to visible light. Next, immunoblotting and immunofluorescence assays were performed to confirm the expression and subcellular localization of the photosensitizers in MSCs. The immunoblotting result showed the constitutional expression of photoreceptors (RHO, RRH, OPN1SW, and OPN4 proteins) from P2, P4, and P6 passages of MSCs (Fig. 1c). As shown in Fig. 1d, the immunoblotting assay showed the expression of these photoreceptors with blue/red light stimulation. In addition, we performed an immunofluorescence assay and confirmed the presence of these photosensitizers in MSCs. (Fig. 1e). Collectively, these data indicated MSCs are sensitive to blue/red light illumination.

### Monochromatic blue light enhances the proliferation of HUVECs co-cultured with MSCs

**Fig. 2:**
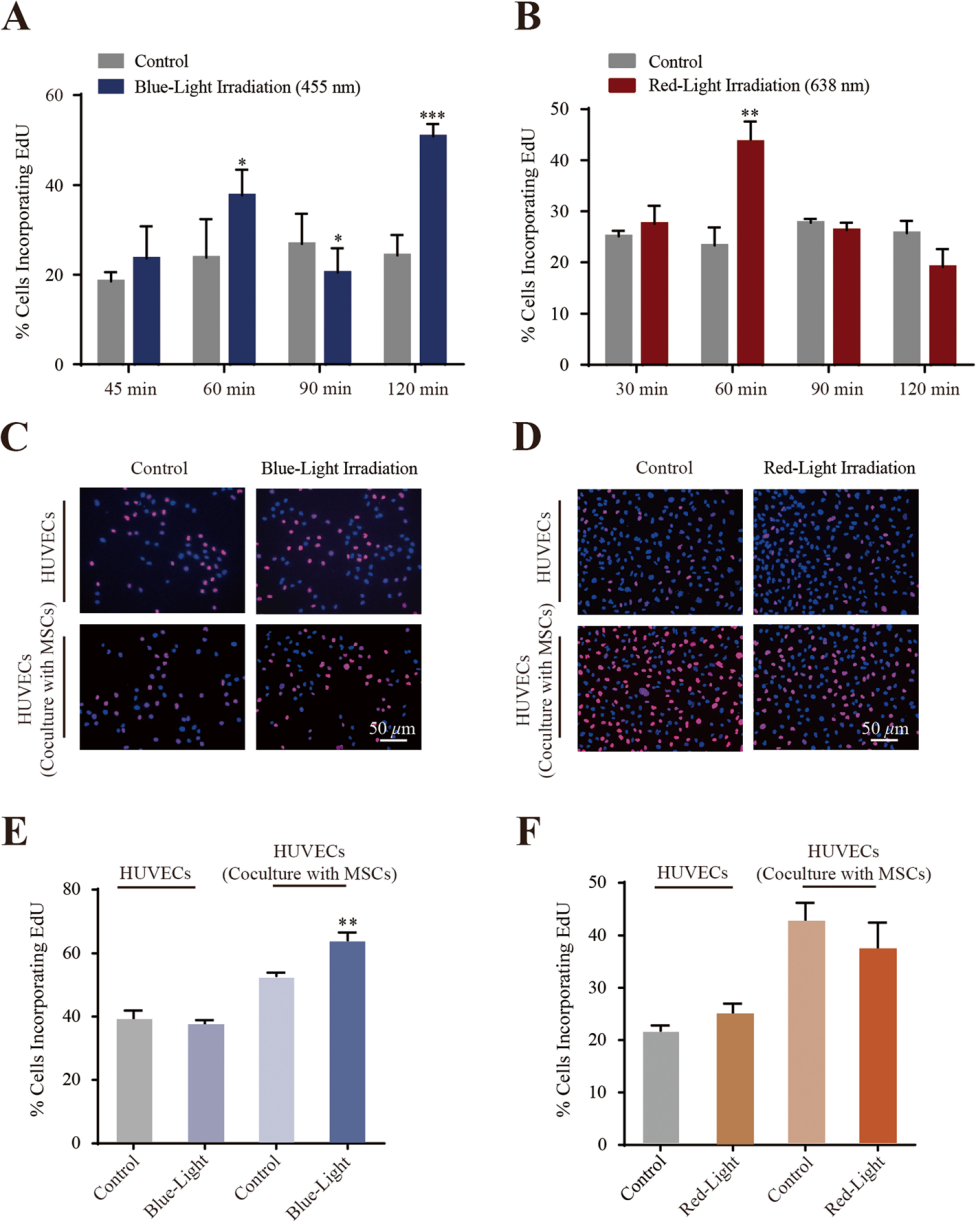
Monochromatic blue light irradiation promoted MSC and HUVEC proliferation in a co-culture system. **a**, **b** Quantitation data of MSCs proliferation under blue/red light illumination. *P < 0.05; **P < 0.01, ***P < 0.001. **c**, **d** Photoimages of EdU incorporation assay of HUVECs co-cultured with/without MSCs under blue and red light exposure, respectively. **e**, **f** Quantitation data of HUVEC proliferation under blue and red light illumination, respectively. **P < 0.01

We measured the effect of monochromatic light treatment by measuring EdU incorporation. MSCs were irradiated for 45, 60, 90, or 120 min daily for three consecutive days with power densities of 300 μW/cm^2^. As shown in Fig. 2a, proliferation was enhanced in the cells treated with blue light for 60 min daily for 3 days (37.67 ± 0.06% EdU-positive) compared to the time-matched dark control cells (23.79 ± 0.09% EdU-positive). Exposure with blue light for 120 min, 50.89 ± 0.03% of cells were EdU-positive in comparison to 24.25 ± 0.05% in control cells. In contrast, proliferation was inhibited in the cells treated with blue light for 90 min daily for 3 days (20.41 ± 0.05% EdU-positive) compared to the time-matched dark control cells (26.81 ± 0.07% EdU-positive). To further investigate the role of the wavelength size in such a monochromatic-induced cell self-renewal, blue light (455 nm) was replaced in the experiment with red light (638 nm), whose wavelength spectrum does not overlap with that of the blue light. Under the same illumination conditions as the blue light irradiation, red light only affected the proliferation of MSCs following an irradiation time of 60 min daily for three consecutive days (Fig. 2b). The findings indicated “60 min daily irradiation time” has better availability and consistency. In addition, light irradiation did not significantly raise the local temperature after the illumination. On average, the culture medium temperature under LED irradiation was less than 0.1 °C higher than that of the dark control group. Collectively, these data indicated that monochromatic blue light is more effective in triggering MSC proliferation than red light.

To further verify the constitutionally expressed photoreceptor that directed the blue light irradiation-induced cell proliferation, *OPN4* was selected and analyzed using RNA interference. OPN4 (Melanopsin), a non-visual opsin best characterized in intrinsically photo-sensitive retinal ganglion cells [20], forms a pigment maximally sensitive to approximately 450–480-nm blue light [21]. Therefore, we transfected MSCs with the RNA interference (control or si*OPN4*) and observed corresponding change of OPN4 protein in the cells (Additional file 2: Figure S1A). We then conducted EdU incorporation assays using these cell lines, and the results showed decreased EdU-positive in *OPN4* RNAi cells compared with the irradiated cells (Additional file 2: Figure S1B and S1C). It will be interesting to systemically investigate the specific role of photoreceptors that direct light-induced cell change in further studies; however, in the present study, we focused only on the investigation of the influence of blue light exposure on the proangiogenic capability of exosomes secreted by MSCs.

Neovascularization is one of the most important therapeutic mechanisms in stem cell-mediated tissue regeneration. We first used the direct contact method of hUC-MSCs and HUVECs in a co-culture system. The effects of monochromatic blue light on proliferation of co-cultured or non-co-cultured ECs were examined using EdU incorporation assays. As shown in Fig 2c–f, both monochromatic blue light and red light could not accelerate HUVEC proliferation compared to control cells. Interestingly, the proliferation of HUVECs co-cultured with MSCs was enhanced under blue light exposure compared to the control cells. However, the number of HUVECs was not increased when treated with red light exposure compared to the dark control, demonstrating that monochromatic blue light is more effective in triggering the proliferation of co-cultured HUVECs.

### Monochromatic blue light accelerates HUVEC migration and vessel formation co-cultured with MSCs

**Fig. 3:**
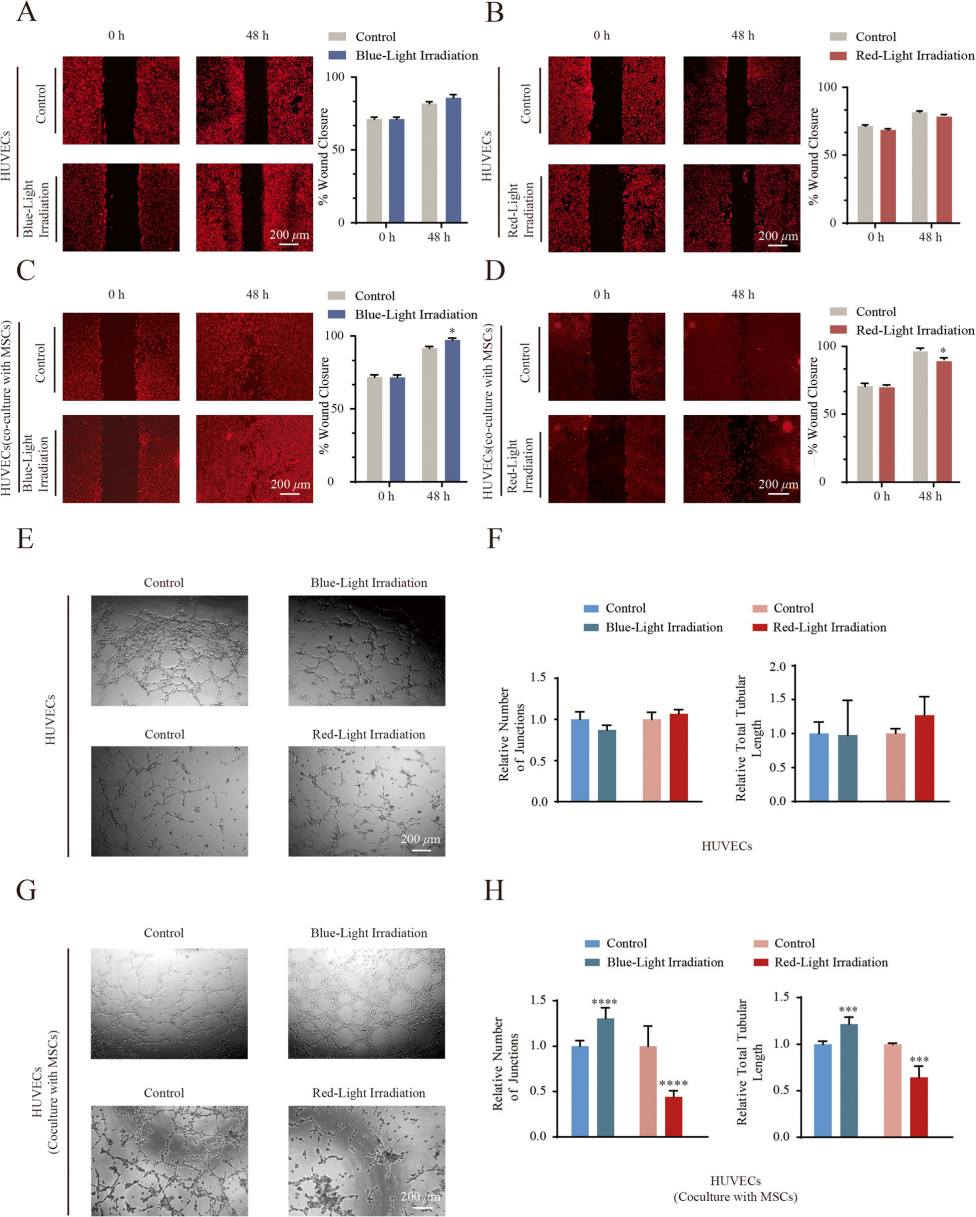
Monochromatic light differentially modulates the migration and vessel formation of HUVECs co-cultured with MSCs**. a**, **b** Photoimages and quantitation data of scratch assay of HUVECs treated with blue/red light, respectively. Photoimages are representative data on 48 h from three independent experiments. Compiled data of migration rates from three independent experiments is shown. Columns, mean; Bars, ± SD. **c**, **d** Photoimages and quantitation data of scratch assay of HUVECs co-cultured with MSCs that were treated with blue/red light, respectively. Photoimages are representative data on 48 h from three independent experiments. Compiled data of migration rates from three independent experiments is shown. Columns, mean; Bars, ± SD, *P < 0.05. **e** Photoimages of vessel formation of HUVECs under monochromatic blue or red light exposure. Results are representative data from three independent experiments. **f** Tubular lengths and number of junctions assay of HUVECs under light exposure. Results are presented as means ± SD of three independent experiments. **g** Photoimages of vessel formation of HUVECs co-cultured with MSCs under monochromatic blue or red light exposure. Results are representative data from three independent experiments. **h** Tubular lengths and number of junctions assay of HUVECs co-cultured with MSCs under monochromatic blue and red light exposure. Results are presented as means ± SD of three independent experiments. ***P < 0.001; ****P < 0.0001

To further determine whether the monochromatic blue light stimulates migration of HUVECs, an in vitro wound closure (scratch assay) test was performed to monitor the rate of cell migration. As shown in Fig. 3a, b and Additional file 3: Figure S2A-S2D), monochromatic blue/red light could not accelerate the rates the HUVEC migration. After 24 h of co-culture with MSCs, the migration distance was significantly greater in HUVECs illuminated with blue light compared with those illuminated with red light or the dark control group. As shown in Fig. 3c and Additional file 3: Figure S2E, S2F, monochromatic blue light accelerated the rates of cell migration compared with control cells. However, the migration rate of HUVECs under red light exposure was lower than dark control cells (Fig. 3d and Additional file 3: Figure S2*G*, S2*H*).

The ability of monochromatic blue light to promote angiogenesis in vitro was evaluated by using an endothelial tube formation assay. As shown in Fig. 3e and f, monochromatic blue/red light could not enhance the blood vessel tube formation of HUVECs without being co-cultured with MSCs. After being exposed to 455-nm blue light for 60 min daily over three consecutive days, blood vessel tube formation was significantly increased in HUVECs co-cultured with MSCs (Fig. 3g, h, *blue bars*). However, both the blood vessel number and the tubular length were significantly decreased in HUVECs treated with red light illumination (Fig. 3g, h, *red bars*). Collectively, these data indicate that monochromatic blue light can promote migration and stimulate the in vitro angiogenic potential of HUVECs co-cultured with MSCs.

### In vivo angiogenesis is promoted by exosomes from MSCs upon blue light stimulation

**Fig. 4:**
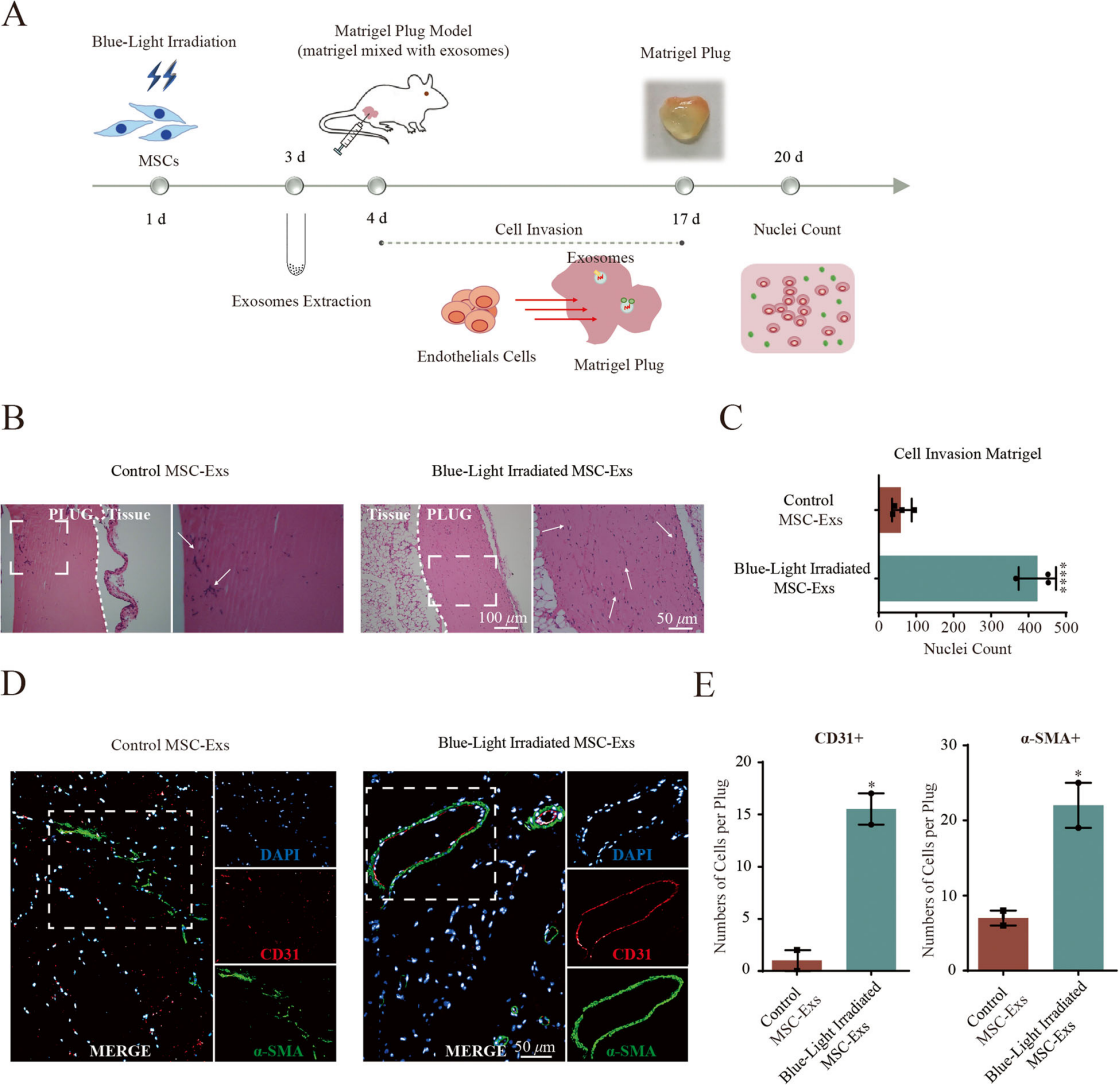
Blue light-treated MSC-Exs enhance the influx of vascular cells into the matrigel plug in mice. **a** In vivo experimental scheme for 455-nm blue light stimulation of the MSC exosomes in matrigel plug assay in BALB/C-nu mice. **b** Representative pictures of in vivo matrigel plug assay lead to cell infiltration into the matrigel plug with MSC exosomes with/without blue light irradiation. The bottom photos were amplification of the box on the above photos. **c** Quantitation data of cell infiltration in the presence of blue light-stimulated MSC-Exs compared to control MSC-Exs matrigel plugs. Results are presented as means ± SD of three independent experiments. ****P < 0.0001. **d** Representative pictures of CD31 and α-SMA staining in matrigel plugs with blue light-stimulated MSC-Exs and control MSC-Exs. **e** Quantitation data of CD31^+^ and α-SMA^+^ cells demonstrated that more endothelial cells infiltrate the matrigel plug in the presence of blue light-stimulated MSC-Exs. Results are presented as means ± SD of three independent experiments. *P < 0.05

Next, we investigated the proangiogenic capacity of MSC-derived exosomes under blue light exposure in vivo using a matrigel plug assay, as shown in Fig. 4a. Both the infiltration of cells and formation of blood vessels were analyzed. As shown in Fig. 4b and c, blue light stimulation of the MSC-derived exosomes in the matrigel plug before subcutaneous implantation led to a 7.0-fold increase of infiltrating cells when compared to MSC-Exs without illumination (an average of 424 MSC-Exs with illumination versus 59 MSC-Exs without illumination, *P* < 0.0001).

To identify formation of blood vessels, the cells in the matrigel plug were further stained for CD31 and α-SMA (Fig. 4d). The number of CD31^+^- and α-SMA^+^-infiltrating cells in exosome-containing plugs was increased 7.8- and 3.1-fold upon blue light stimulation compared to control exosomes without illumination (Fig. 4d, e). Additional analysis showed colocalization of α-SMA and CD31, which suggests the formation of mature vessels. In summary, exosomes from MSCs are capable of increasing overall cell invasion in a matrigel plug and inducing the formation of functional vessels upon 455-nm blue light stimulation, supporting their proangiogenic capability.

### MSC-derived exosomes promote angiogenesis in a cutaneous burn model upon blue light irradiation

**Fig. 5:**
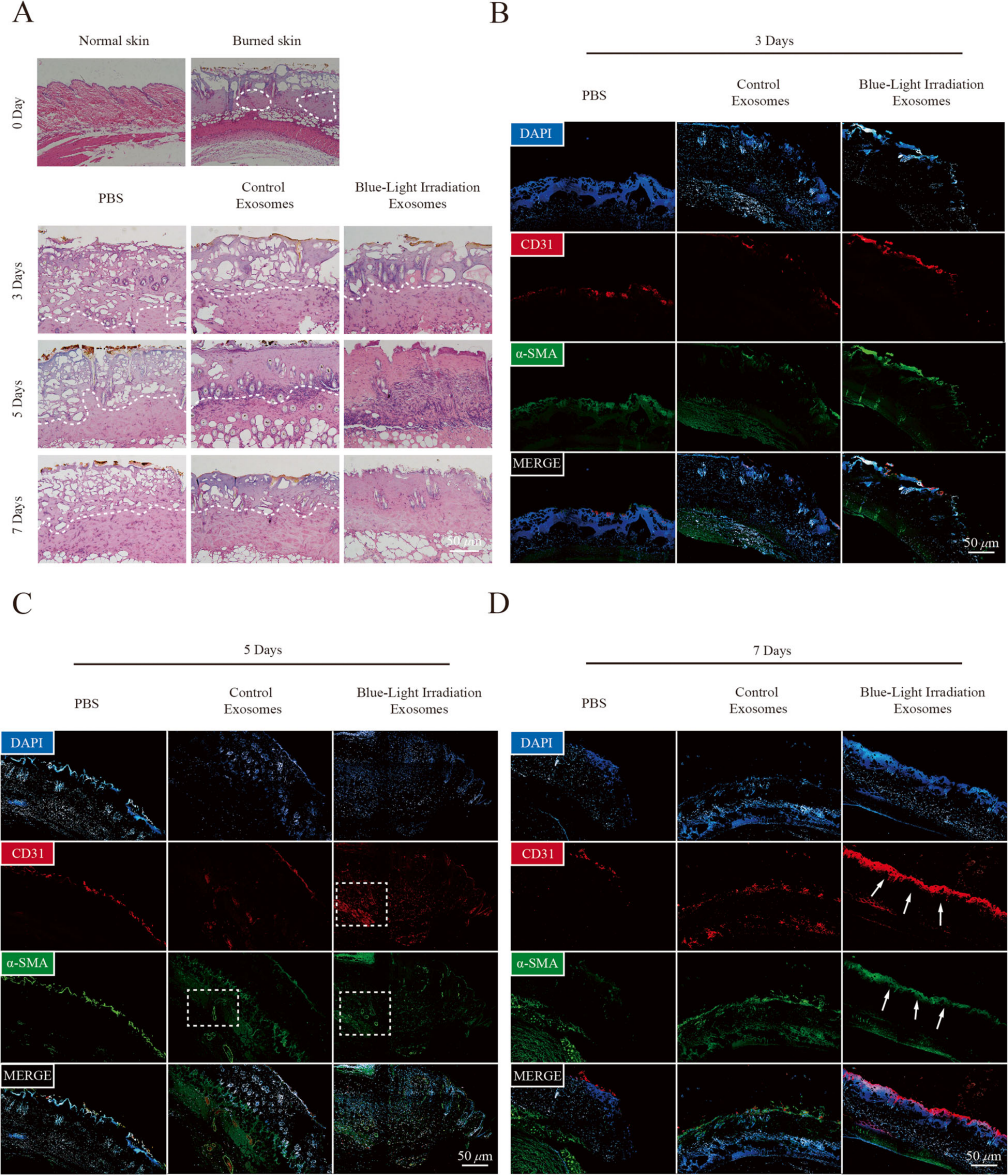
MSC exosomes upon blue light stimulation promoted angiogenesis in a cutaneous burn model in vivo. **a** Representative cross section of wound histological images with H&E staining at 0, 3, 5, and 7 days after treatment with PBS, MSC-Exs, or MSC-Exs stimulated with blue light. **b**–**d** Representative cross section of wound histological images with CD31 and α-SMA immunofluorescence staining at 3, 5, and 7 days as indicated treatments

To confirm the effects of blue light-treated exosomes on angiogenesis in vivo, we established a skin-deep, second-degree burn model in mice. The results of histological evaluation of wounds at 3, 5, and 7 days after infusion showed that the number of epidermal and dermal cells significantly increased in blue light-treated exosome wounds compared with control exosome wounds, whereas wounds treated with PBS remained in a second-degree burn injury state (Fig. 5a). Furthermore, the results of immunofluorescence staining for vascular endothelial cell markers CD31 and α-SMA showed that the blue light-treated exosome group had more CD31^+^ and α-SMA^+^ cells in the wound area than the control exosome group at 3, 5, and 7 days post-infusion (Fig. 5b–d). Altogether, these results revealed that 455-nm blue light irradiation can stimulate the proangiogenic ability of MSC-Exs in vivo*.*

### Monochromatic blue light promotes MiR-135b- and MiR-499a-induced MEF2C signaling in MSC-derived exosomes

**Fig. 6:**
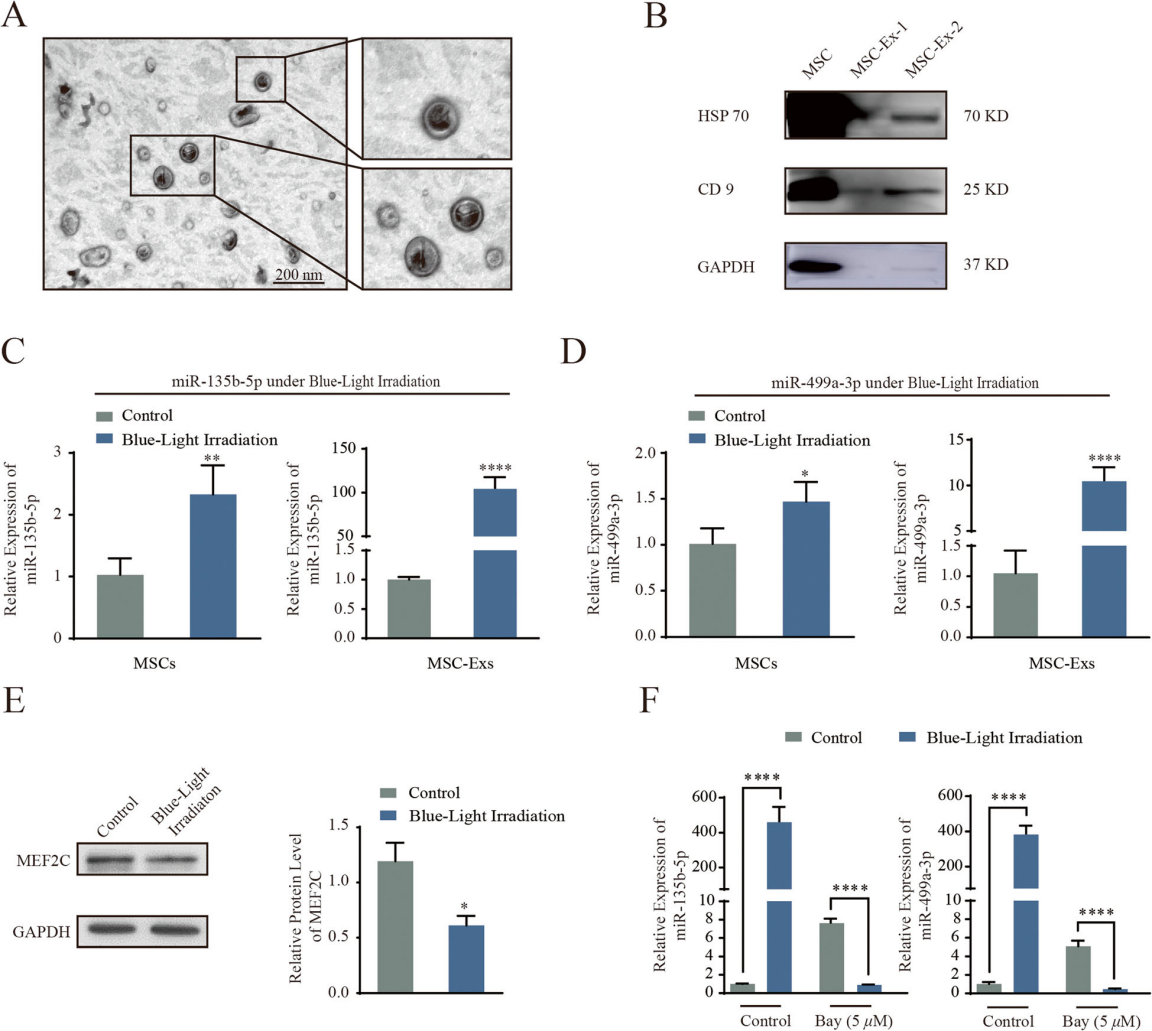
Blue light increased miRNAs (MiR-135b and MiR-499a)-MEF2C signaling in MSC-Exs. **a** Transmission electron microscopy analysis of MSC-derived exosomes. **b** Western blot analysis of the expression of exosomes enriched markers HSP 70 and CD 9 on the exosomes. **c, d** qRT-PCR for miR-135b-5p and miR-499a-3p derived from MSCs or MSC-Exs as indicated treatments. The *P* values were calculated by two-sided Student’s *t* test. **P < 0.01; ****P < 0.0001. **e** Western blot analysis of MEF2C expression in MSCs as indicated treatments. Compiled data from three independent experiments is shown. Columns, mean; bars, ± SD; *P < 0.05. **f** qRT-PCR for miR-135b-5p and miR-499a-3p derived from MSCs or MSC-Exs as indicated treatments. The *P* values were calculated by two-sided Student’s *t* test. ****P < 0.0001

The morphology of purified MSC-derived exosomes was observed by transmission electron microscopy. As shown in Fig. 6a, the exosomes had a characteristic saucer-like shape that was limited by a lipid bilayer with a diameter ranging from 30 to 100 nm. As shown in Fig. 6b, immunoblotting results confirmed that MSC-Exs expressed exosomal markers such as HSP70 and CD9.

We then sought to identify the mechanism that elicits the enhanced proangiogenic ability of the blue light-stimulated MSC-Exs. Previously, we have shown that serum exosome-derived miR-135b-5p and miR-499a-3p are taken up by ECs and jointly repress *MEF2C* gene xpression [16]. Given the enhanced proliferation and migration demonstrated in ECs in response to miRNA-mediated *MEF2C* suppression [16, 22, 23], we thus compared the level of expression of the two miRNAs in MSC-derived exosomes with and without blue light irradiation. As shown in Fig. 6c and d, both miRNAs were found to be significantly upregulated in blue light-treated MSCs and MSC-Exs compared to controls. Our results showed a 2.3-fold and 104.1-fold increase in miR-135b-5p expression upon blue light stimulation in MSCs and MSC-Exs, respectively. Our results also showed a 1.4-fold and 9.9-fold increase in miR-499a-3p expression upon blue light stimulation in MSCs and MSC-Exs, respectively. Correspondingly, MEF2C protein expression was simultaneously decreased following irradiation with 455-nm blue light (Fig. 6e). In addition, we added BAY 11-7082 (an NF-κB inhibitor) and observed that a reduction in NF-κB caused a decrease in miR-135b-5p and miR-499a-3p levels (Fig. 6f). Collectively, our results indicate that the elevated miR-135b-5p and miR-499a-3p levels under blue light exposure increased proangiogenic activity in MSC-Exs.

## Discussion

More evidence is emerging that MSC-derived exosomes are promising options for producing the beneficial effects of proangiogenic therapy in tissue regeneration, possibly because MSC exosomes have been found to elicit very low levels of immunogenicity [24]. In addition, strategic manipulation of the important miRNAs [25] or proteins [26] in exosomes could improve their therapeutic effect. Our previous study has demonstrated that exosomes derived from serum contain miR-135b-5p and miR-499a-3p that work together to repress MEF2C gene expression and promote the proliferation and migration of HUVECs [16]. In this study, exosomes were obtained from blue light-treated hUC-MSCs, and this exposure increased proangiogenic potential by stimulating ECs. In particular, two miRNAs, miR-135b-5p and miR-499a-3p, in the exosomes, were found to be highly expressed in blue light-treated exosomes, which are capable of eliciting capillary formation both in vitro and in vivo.

Therapeutic potential of stem cell-derived exosomes for tissue repair [27] and regeneration [28] has been reported by several preclinical experiments. Exosomes are smaller, less complex, and immunogenic compared to the corresponding stem cells that produce them [29]. For example, exosomes derived from various stem cells have recently been found to act as effective regulators of angiogenesis where they can improve heart function by participating in cardiac protection and repair [30]. Our exploration to assess the wavelength-dependent modulation of angiogenesis capacity of MSC-derived exosomes by light exposure revealed interesting and encouraging results. We showed that 455-nm blue light exposure is effective in enhancing the angiogenesis ability of exosomes compared with 638-nm red light or darkness.

**Fig. 7:**
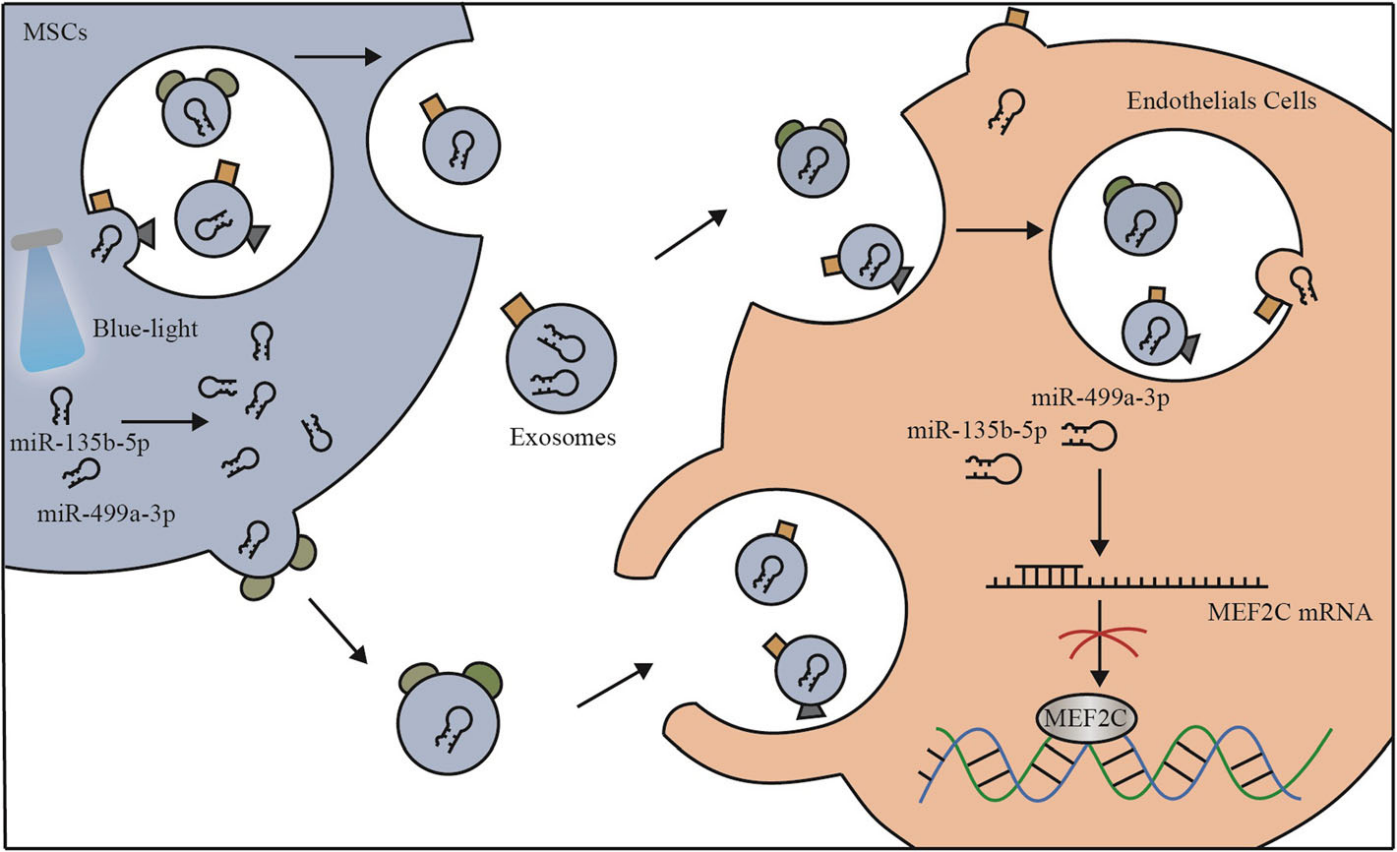
Putative mechanism by which blue light increases the two miRNAs to activate the ECs. See the “Discussion” section for further details

Exosomes derived from MSCs are enriched in mRNAs or miRNAs and are representative of their cellular origin [31]. For example, miR-15a, miR-15b, and miR-16 are able to inhibit the expression of CXCL1 and mediate the therapeutic effects of exosomes from hUC-MSCs in models of acute kidney injury [32]. Here, we showed that MSC-derived exosomes have a similar effect and this effect could be modulated by light exposure in a wavelength-dependent manner. In addition to these stimulatory effects observed in vitro, angiogenesis is also enhanced in vivo upon blue light-treated exosome stimulation. We also observed an increase in vascular cell invasion and blood-filled capillary formation in vivo when exosomes were applied in both matrigel plug and skin wound models. When attempting to understand the mechanism underlying the proangiogenic effect of MSC-Exs stimulated by monochromatic blue light exposure, we observed that activation of the endothelial cells corresponded with an increase in both miR-135b-5p and miR-499a-3p (Fig. 7).

There are several other significant proteins and miRNAs known to influence angiogenesis using exosomes from MSCs [33] . It would be interesting to study the regulation by other proangiogenic proteins or miRNAs in future studies. Thus, investigating detailed target pathways and their functional roles is necessary to offer insights into the universal guidelines of photomodulation. However, in the present study, we focused on miR-135b-5p and miR-499a-3p to investigate their potential roles in the release of exosomes induced by 455-nm blue light. Furthermore, there is still a debate ongoing regarding the use of a high concentration of exosomes as a single treatment versus the continuous release of exosomes from MSCs. In our current study, we have only focused on the angiogenic effect of blue light-treated exosomes from MSCS with established in vitro and in vivo models. Thus, further investigation into the manner of modulation is needed to delineate the full range of physiological functions of MSC exosomes.

## Conclusions

Future clinical therapies using MSC-derived exosomes are very promising due to their proangiogenic ability in tissue repair, possibly because MSC exosomes have been found to elicit very little immunogenicity [1]. Our results here demonstrate that blue light illumination of exosomes can improve their therapeutic effects by increasing their proangiogenic ability. Elevated levels of miR-135b-5p and miR-499a-3p led to increased proangiogenic potential by stimulating ECs. These findings may reveal important insights into the role of light exposure in the use of MSC-derived exosomes in tissue regeneration and, furthermore, suggest that miR-135-5p and miR-499a-3p may serve as a novel therapeutic target for therapy. However, it is likely that many other factors are involved in exosome-dependent angiogenesis. Therefore, investigation into additional factors and their functional roles is necessary to offer insights into the role of blue light illumination in tissue repair and regeneration.

## Supplementary information

**Additional file1:** **Table S1.** Primers for Real-time PCR.

**Additional file 2: Figure S1.** OPN4 served as photoreceptors response to blue light irradiation-induced MSCs proliferation. (A) MSCs were transfected with siRNA oligonucleotide silencing *OPN4*. Equivalent amounts (30 *μ*g) of whole-cell lysates were separated by SDS-PAGE and analyzed by immunoblotting with antibodies specific for the indicated proteins. (B) Representative photoimages of EdU incorporation of MSCs transfected with si*OPN4* under blue light exposure. (C) Quantitation data of MSCs proliferation at the indicated treatments from three independent experiments. *Columns*, mean; *Bars*, ±S.D.; *P < 0.05.

**Additional file 3:** **Figure S2.** Monochromatic light differentially modulates the migration of HUVECs co-cultured with MSCs. (A-D) Photoimages and quantitation data of scratch assay of HUVECs only treated with blue/red light, respectively. Photoimages are representative data on 0, 12, 24 and 48 hours from three independent experiments. Compiled data of migration rates from three independent experiments is shown. Columns, mean; Bars, ± SD. (E-H) Photoimages and quantitation data of scratch assay of HUVECs co-cultured with MSCs that were treated with blue/red light, respectively. Photoimages are representative data on 0, 12, 24 48 hours from three independent experiments. Compiled data of migration rates from three independent experiments is shown. Columns, mean; Bars, ± SD, *P < 0.05.

## Data Availability

All data generated or analyzed during this study are included in this published article and its additional files.

## Abbreviations

MSCs: Mesenchymal stem cells
hUC-MSCs: Human umbilical cord mesenchymal stem cells
HUVECs: Human umbilical vein endothelial cells
MSC-Exs: MSC-derived exosomes
miR: MicroRNA
ECs: Endothelial cells
MEF2C: Myocyte enhancer factor 2C
EdU: 5-Ethynyl-2′- deoxyuridine
LED: Light-emitting diode
