# Supplementary material for: Exposure to blue light stimulates the proangiogenic capability of exosomes derived from human umbilical cord mesenchymal stem cells
Source: Stem Cell Res Ther. 2019 Nov 28;10:358. doi: 10.1186/s13287-019-1472-x (PMC6883639; doi:10.1186/s13287-019-1472-x)
Supplement: Supplementary file 1 — Additional file1: Table S1. Primers for Real-time PCR. [file 13287_2019_1472_MOESM1_ESM.docx]

**Supplementary Table S1. Primers for Real-time PCR**

| Gene | Gene Title | Primer Sequence Gene | Product  (bp) |
| --- | --- | --- | --- |
| *RRH* | retinal pigment epithelium-derived rhodopsin homolog | F: 5’-ACCACCAACACTTACATCGG-3’  R: 5’-TAGCACCAGTAGGATCTGGG-3’ | 112 |
| *RGR* | retinal G protein coupled receptor | F: 5’-GTTGGGGTCACTACGACTATGA-3’  R: 5’-GGCATGGCGAAGTTGAAGAA-3’ | 118 |
| *OPN1SW* | short-wave-sensitive opsin1  (cone pigment) | F: 5’-ATGGGCCTCAGTACCACATT-3’  R: 5’-GCAACTTTTTGTAGCGCAGT-3’ | 132 |
| *OPN1MW* | medium-wave-sensitive opsin1  (cone pigment) | F: 5’-CATCCGCAGGACAGCTATGAG-3’  R: 5’-GGAGCGATGTGGTAATTCGGG-3’ | 107 |
| *OPN1LW* | long-wave-sensitive opsin1  (cone pigment) | F: 5’-CCCACTCGCTATCATCATGCT-3’  R: 5’-CAGTACGCAAAGATCATCACCA-3’ | 144 |
| *RHO* | rhodopsin (opsin2, rod pigment) | F: 5’-ACAGGATGCAATTTGGAGGGC-3’  R: 5’-GCTCATGGGCTTACACACCA-3’ | 111 |
| *OPN3* | opsin3 (encephalopsin, panopsin) | F: 5’-TCAGTGCACAATGGCTAGAG-3’  R: 5’-GCGGTTCCCCGAGTACAT-3’ | 135 |
| *OPN4* | opsin4 (melanopsin) | F: 5’-CTGGATCTCCATACGGAGGC-3’  R: 5’-GTAGAGGGACCGCCCATTT-3’ | 121 |
| *OPN5* | opsin5 | F: 5’-TTGGGAAGCGGATTTAGTGG-3’  R: 5’-TTATTTCAGCGGGTCTCAGC-3’ | 125 |
