# Supplementary figures and images for: Exposure to blue light stimulates the proangiogenic capability of exosomes derived from human umbilical cord mesenchymal stem cells
Source: Stem Cell Res Ther. 2019 Nov 28;10:358. doi: 10.1186/s13287-019-1472-x (PMC6883639; doi:10.1186/s13287-019-1472-x)

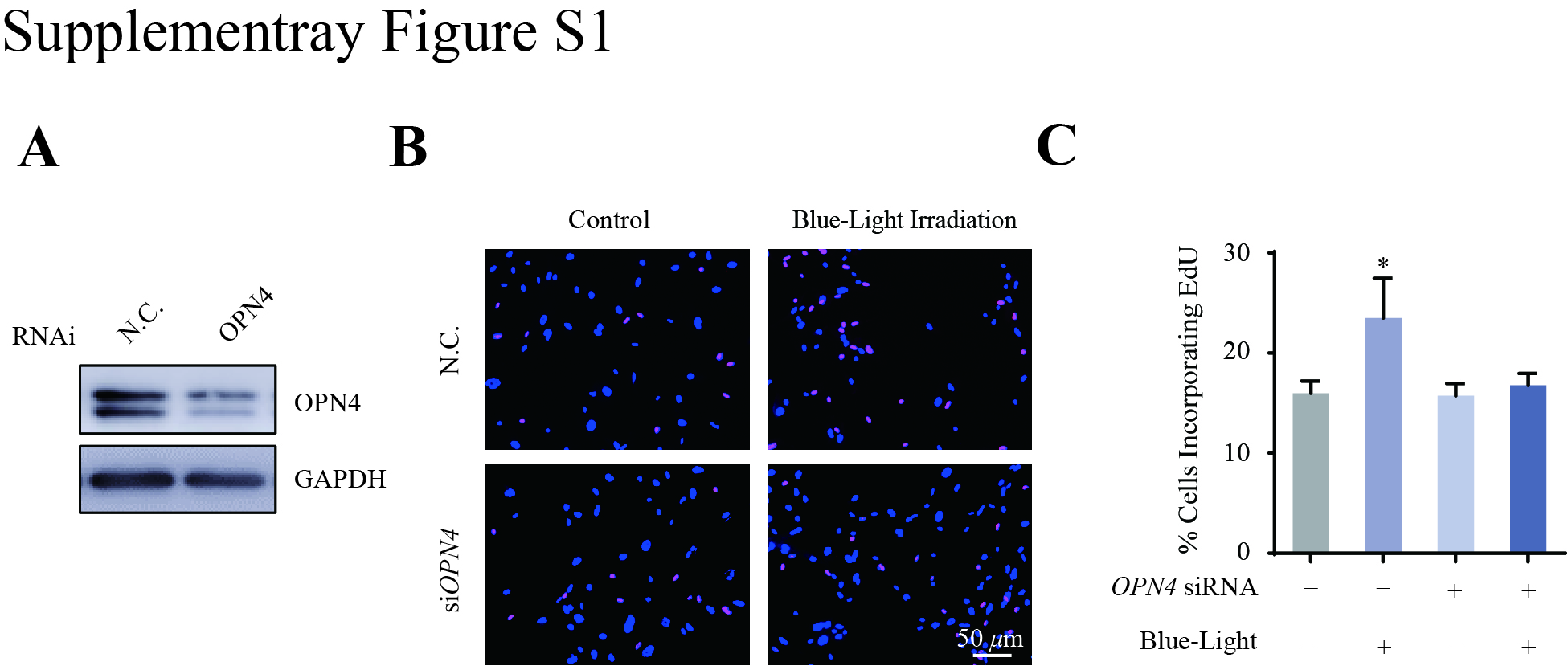

Supplement: Supplementary file 2 — Additional file 2: Figure S1. OPN4 served as photoreceptors response to blue light irradiation-induced MSCs proliferation. (A) MSCs were transfected with siRNA oligonucleotide silencing OPN4. Equivalent amounts (30 μg) of whole-cell lysates were separated by SDS-PAGE and analyzed by immunoblotting with antibodies specific for the indicated proteins. (B) Representative photoimages of EdU incorporation of MSCs transfected with siOPN4 under blue light exposure. (C) Quantitation data of MSCs proliferation at the indicated treatments from three independent experiments. Columns, mean; Bars, ±S.D.; *P < 0.05. [file 13287_2019_1472_MOESM2_ESM.jpg]

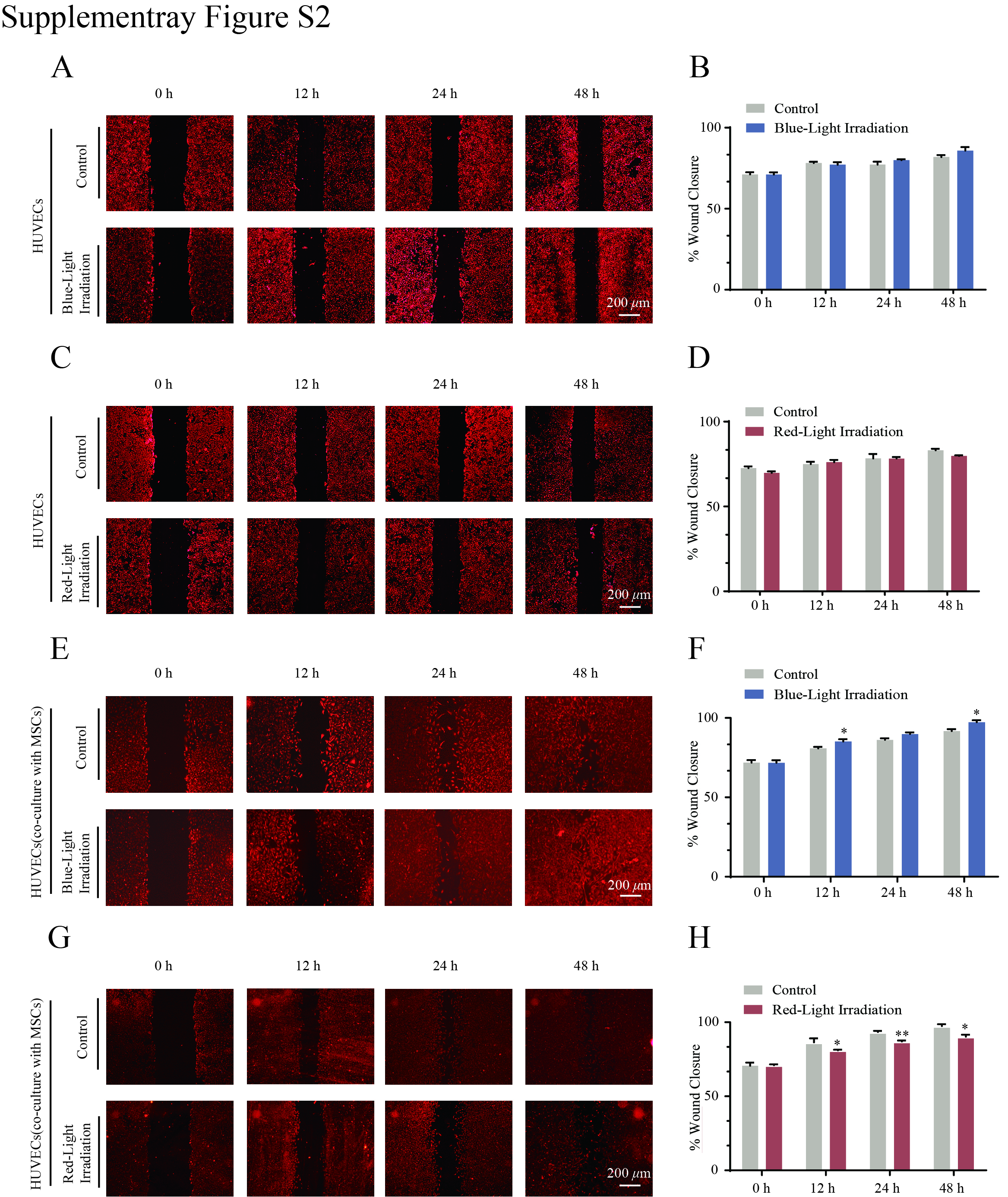

Supplement: Supplementary file 3 — Additional file 3: Figure S2. Monochromatic light differentially modulates the migration of HUVECs co-cultured with MSCs. (A-D) Photoimages and quantitation data of scratch assay of HUVECs only treated with blue/red light, respectively. Photoimages are representative data on 0, 12, 24 and 48 hours from three independent experiments. Compiled data of migration rates from three independent experiments is shown. Columns, mean; Bars, ± SD. (E-H) Photoimages and quantitation data of scratch assay of HUVECs co-cultured with MSCs that were treated with blue/red light, respectively. Photoimages are representative data on 0, 12, 24 48 hours from three independent experiments. Compiled data of migration rates from three independent experiments is shown. Columns, mean; Bars, ± SD, *P < 0.05. [file 13287_2019_1472_MOESM3_ESM.jpg]
